# The impact of COVID-19 on an employee assistance programme in a multinational insurance organisation: Considerations for the future

**DOI:** 10.4102/sajip.v47i0.1863

**Published:** 2021-09-27

**Authors:** Dieter Veldsman, Ninette van Aarde

**Affiliations:** 1 Department of Industrial Psychology and People Management, College of Economics and Finance, University of Johannesburg, Johannesburg, South Africa; 2 Department of Organisational Effectiveness, Group Human Capital, Momentum Metropolitan, Centurion, South Africa

**Keywords:** employee well-being, employee assistance programmes, wellness, COVID-19, employee value proposition

## Abstract

**Orientation:**

The coronavirus disease 2019 (COVID-19) has led to an increased focus on the effectiveness of employee assistance programmes (EAPs).

**Research purpose:**

To evaluate the impact of COVID-19 on the value, utilisation and scope of an EAP within the South African insurance sector.

**Motivation for the study:**

Higher levels of stress and anxiety experienced by employees because of COVID-19 has necessitated the need to better understand the reasons for EAPs utilisation and its effectiveness within organisations.

**Research approach/design and method:**

The study provided an overview of employee well-being and an overview of the origins and evolution of EAPs. The study utilised thematic analysis to analyse 1002 cases with a sample of *n* = 907, pre-and post-onset of the COVID-19 pandemic.

**Main findings:**

The utilisation of EAPs increased because of COVID-19, yet the reasons for accessing these programmes remained largely consistent before and during COVID-19. At a sub-theme level, the priority of themes differed across the time periods influenced by external context and circumstance.

**Practical/managerial implications:**

The study found a need to clearly define employee well-being and reposition the role of EAPs within the organisation. Organisations need to broaden the scope of EAPs and through continuous education and awareness create an environment where employees feel like they can safely access these services.

**Contribution/value-add:**

The study contributes towards the current literature on employee well-being and providing a perspective on the relevance, value and utilisation of EAPs before and during a pandemic.

## Introduction

The year 2020 will be remembered as the year that changed the world. The coronavirus disease 2019 (COVID-19) pandemic fast-tracked digital transformation and led to a re-evaluation of the relevance of people management priorities and practices (Iida 2020). Employee well-being and employee assistance programmes (EAPs) came under scrutiny as organisations battled to remain productive whilst supporting employees in dealing with unprecedented change. The focus on employee well-being had been gathering traction before COVID-19 because of the changing employee value proposition landscape (De Villiers, 2009; Nangoy, Mursitama, Setiadi, & Pradipto, 2020), yet COVID-19 re-emphasised the importance of employee well-being and the relationship to organisational productivity.

This article explores the utilisation of an EAP over 9 months, 01 January 2020–30 September 2020, to better understand the impact that COVID-19 has had on employees’ needs during this time. Thematic analysis was used in the study to review the utilisation of an EAP within the context of a South African insurance organisation with specific focus on utilisation before and during COVID-19. The article also provides an overview of the literature regarding employee well-being and the origins of EAPs and an overview of the development of the concept and its application in the modern-day organisation.

## Conceptualising and defining well-being and wellness

The rise of the knowledge worker as part of the Third Industrial Revolution led to a renewed focus on human capital practices as critical enablers of business performance (Benson & Brown 2007). Borjas (2016) quoted Swiss novelist Max Frisch in stating, ‘We wanted workers, but people came instead’. This phrase encapsulates the thinking that influenced people practices from the 1960s onwards when the workforce’s wellness became a central theme as an enabler of productivity. Globally, wellness and well-being have become a trillion-dollar industry with a broad focus, ranging from physical to mental and spiritual well-being (Koncul, 2012). The definitions of wellness and well-being, two terms often used interchangeably, stem from the definition of wellness by Dunn (1959) as an integrated method of improvement to maximise the individual potential within a particular environment. It is important to note that this definition does not explicitly refer to a desired state of wellness but rather conceptualises a continuous drive for improvement of a fluctuating state of wellness. In the mid-70s, Hettler (1976) expanded the definition in a model that includes six dimensions of wellness that predominantly focuses on an individual perspective: (1) physical health (body, nutrition, healthy habits), (2) emotional (feelings, emotions, cognitions), (3) employment (work, skills, finances, planning), (4) spiritual (sensitivity, values, self-esteem), (5) social (family, friendships, community) and (6) intellectual (creativity, cognitive, knowledge, independence). This model was later refined to align with the definition provided by the World Health Organisation (WHO) (2010), that wellness is a positive, holistic concept that consists of a multifaceted approach and is not only geared towards the absence of disease but, somewhat, the ability to flourish in different contexts. Su, Tang and Nawijn (2020) described the two dimensions of well-being, which exists in theory: hedonia and eudaimonia. Hedonia is associated with an immediate sensory pleasure, happiness and enjoyment, whilst eudaimonia relates to the result of self-growth and self-actualisation. A notable contribution to the wellness movement is attributed to Travis (1977), who posited that the individual has to take ownership of his or her well-being and made wellness a practical and understandable construct in a publication referred to as *The Wellness Workbook*. This workbook translated the six dimensions articulated by Hettler (1976) into a variety of techniques aimed at building individual self-awareness, to equip individuals to take ownership of their own well-being journeys. With this approach, harmony between body, mind and spirit was seen as the ultimate goal of the well-being journey. Ardell (1979), however, rejected the spiritual influence on holistic well-being and rather focused on body and mind. This approach gathered favour in the 1980s and led to a number of publications in the wellness domain with a strong focus on the physical domain of well-being, largely ignoring mental well-being (Karn, Amarkantak, & Swain, 2017).

## The impact of geography on defining wellness and well-being

Geographical location also seems to influence the understanding of wellness (Sheldon & Bushell, 2009). European definitions of wellness have a broader focus on increased pleasure and ‘feeling well’, giving rise to a ‘wellness tourism’ industry. This industry includes well-being spas and centres offering non-medical treatments, as well as destinations where tourists can unwind and recuperate, with the goal of ‘feeling well’.

This movement expanded to the East, with Asia becoming a strong player in the wellness tourism industry (Heung & Kucukusta, 2013). Commercially, the benefits of well-being on an individual level were popularised by medical aids and insurance companies. Well-being programmes and benefits related to physical wellness and lifestyle choices were conceptualised into reward and loyalty programmes that offer participants benefits if they demonstrate healthy lifestyle choices over time (Aon, 2017). The wearable fitness industry further advanced this perspective by enabling individuals to monitor, track and evaluate their exercise patterns, heartbeat and related metrics, which can be utilised to influence medical aid premiums and life insurance risk profiles. In turn, these programmes provide a multitude of data that can be used to explore and monitor general well-being across various dimensions.

The given overview indicates that wellness and well-being are not clearly defined and the difference between the terms is not clear. The lack of clarity is problematic as it leads to limiting views and understanding of these concepts. The literature indicates significant differences between the two terms. Wellness seems to be more associated with physical health, emphasising the adoption of positive health practices and lifestyle choices, with a firm root in medical sciences. In this definition, mental wellness is seen predominantly as a reactive approach to deal with traumatic events instead of a proactive perspective that encourages human flourishing. The term is used in the populist literature and is largely utilised for commercial purposes as part of the wellness tourism industry and to manage risk in the medical aid and insurance domains. According to this definition, feeling well is the goal and this can be attained mainly through a healthy lifestyle. Well-being seems to be a term with its roots in the dimensions identified by Hettler (1976) in terms of mental, financial, physical, spiritual and emotional well-being. Well-being also seems to be associated with a continuous journey towards a state rather than reaching a particular goal. In this definition, there is a strong focus on body, mind and spirit as three parts of the equation and all are equally important to achieve equilibrium. This definition seems to be more consistently used in academic literature and has strong origins in psychology and medical sciences. This holistic view of well-being has entered the public domain over recent years, with mental well-being becoming increasingly crucial in the global health narrative to destigmatise mental health conditions in the organisational context.

Table 1 summarises the similarities and differences between wellness and well-being from the literature.

**TABLE 1:** Wellness and well-being: Similarities and differences.

Wellness		Well-being
Strong focus on physical wellness with strong origins in medical sciences	Origin	Strong focus on mind, body, and spirit, with strong origins in both psychology and medical sciences
Goal of wellness is to make informed lifestyle choices to feel well, which leads to healthier habits	Outcome	Level of well-being exists on a continuum between a high level of well-being and illness, with individuals continuously moving along this continuum
Responsibility lies with individuals to manage their own wellness through the choices they make	Responsibility	Responsibility lies with individuals to manage their own well-being; as such, self-awareness is important to better understand the specific aspects of one’s own well-being journey

*Source*: Adapted from Els and De la Rey 2006; Thal and Hudson 2006; Smith and Reid 2018; McMahon, Williams and Tapsell 2010; Hattie, Myers and Sweeney 2004; Macdonald 2005; Kaliatkaitė and Bulotaitė 2014

Note: Please see the full reference list of the article: Veldsman, D., & Van Aarde, N. (2021). The impact of COVID-19 on an employee assistance programme in a multinational insurance organisation: Considerations for the future. SA Journal of Industrial Psychology/SA Tydskrif vir Bedryfsielkunde, 47(0), a1863. https://doi.org/10.4102/sajip.v47i0.1863, for more information.

This article defines well-being as an individual level construct described through six dimensions that refers to physical, emotional, employment, spiritual, social and intellectual needs. This is in line with the arguments made by Hettler (1976) and assumed that wellness is an important sub-dimension of overall well-being largely focused on the physical dimension.

## The scope and contribution of employee assistance programmes in organisations

The Canadian Centre for Occupational Health and Safety (2020) defines EAPs as a programme provided by an employer or service provider with a focus on addressing the well-being of employees that might be impacting their performance. Even though the purpose, objective and scope of EAPs have changed vastly over time, several beliefs and misconceptions based on the origins of these programmes prevail. Trice and Schonbrunn (1981) stated that EAPs were put in place as a result of social problems, such as alcohol and substance abuse, becoming prevalent in the 1940s post-World War II era. Whilst the intent of employers was to improve the well-being of workers, there was also a strong commercial consideration. The cost of replacing workers had skyrocketed and outweighed the cost associated with implementing support programmes as opposed to disciplinary procedures. Today, most large employers provide some form of assistance to employees in the form of either an in-house or an outsourced EAP (Mattke et al., 2013). The contents of these programmes differ, yet most contain components of mental well-being through the provision of psychological support services and referral methods to provide support in overcoming substance abuse. In general, however, these programmes are designed to deal with issues that have already occurred and are largely seen as a reactive response to impaired well-being. It is important to note the focus of these programmes; whilst they provide assistance to individuals in a time of need, the programmes are designed to do so with a strong focus on optimising employee productivity within a specific employer context, and, as such, are usually short term in nature. EAPs service offerings have been expanded, and, today, most EAPs contain psychological services, financial planning, and support services (including access to short-term financial loans), legal support, trauma counselling, career guidance and a focus on physical wellness through sports clubs and related rewards. During the COVID-19 pandemic organisations have further expanded their offering to include more counselling sessions for employees and their families either free or at a reduced cost, online mental health resources, well-being coaching sessions, more flexible work schedules to take time to recharge, online resources such as webinars, articles, blogs and meditation and mindfulness applications (Ward, 2020). The EAP is usually managed by the human resources department and is predominantly positioned as part of the value offering that responsible employers provide to their employees, executed through line management (Blandin De Chalain 2020).

Employee assistance programmes can be differentiated from traditional mental health programmes through the presence of the following elements (Attride, 2019; Sonnenstuhl & Trice, 2018):

- **Work focus:** The main reason for the implementation of an EAP is to support employees to be productive in the work context.
- **Manager training and involvement:** Given that managers’ positioning enables them to notice and identify well-being challenges, an EAP needs to contain a component of manager training to ensure that managers respond effectively and timeously to issues related to employee well-being.
- **Linkages and referrals:** The EAP should inform employees of the support available in their immediate environment and in the broader community and how to access these services.
- **Anonymity and confidentiality:** The EAP needs to be built upon a foundation of trust between employer and employee and ensure the confidential handling of all cases. Even though the employer might carry the cost on behalf of the employee, this does not entitle the employer to access information related to the nature of the EAP intervention.
- **Short-term focus:** Employee assistance programmes provide short-term support and are not designed for long-term intervention.

The past few years have seen the evolution of EAPs in terms of scope and services to support the changing needs of employees and employers. There are however still challenges with regard to the measurement of EAP effectiveness and barriers towards effective utilisation.

## Challenges influencing the effectiveness of employee assistance programmes

The evaluation of EAPs value is typically focused on the alignment of the EAP with the organisation’s philosophy, vendor performance as it relates to the implementation of the programme, effectiveness of interventions in managing functioning and addressing long-term care requirements and cost effectiveness of the programme (Masi, 1997). The Standards for Employee Assistance Programmes in South Africa (EAPA-SA, 2015) provides criteria for evaluation related to international best practice. These standards relate to the establishment and governance of managing an EAP, linking the EAP outcomes with the organisational outcomes, pricing and financial management, resourcing of the EAP, training and ethical standards of practitioners and the implementation of appropriate interventions. In evaluating the effectiveness of an EAP, limited financial resources, availability and accessibility of data and adhering to confidentiality whilst accessing rich information act as barriers to understand the value provided by the programme (Masi, 1997). The effectiveness of EAPs has also come under scrutiny, not only for commercial reasons but also because of incorrect measures being utilised to measure effectiveness. Predominantly, utilisation is seen as a measure of effectiveness, yet because of the reactive nature of most EAP programmes, high levels of utilisation is an indication of significant challenges within the workforce and as such not a good measure of the effectiveness of an EAP (Wills, 2018). Table 2 provides an overview of challenges experienced in implementing EAPs that impact the perception of their value.

**TABLE 2:** Challenges encountered in implementing employee assistance programmes (EAPs).

Challenge	Description
Stigma and stereotyping	Perceptions that EAPs are for employees who are ‘weak’, under-performing or unable to ‘deal with life’
Confidentiality and anonymity	Victimisation of employees who utilise the programme and sharing of information related to personal issues because the employer pays for the service
Organisational culture	An organisational culture that does not promote well-being, but rather celebrates ‘being tough’ and ‘sticking it out’
Cost and value considerations	Inability to quantify programme value versus cost considerations and demonstrate a return on investment
Awareness and knowledge of programmes	Lack of programme awareness and what is on offer amongst employees, especially if programmes are not supported through education campaigns and induction practices
Manager incompetence	Inability of managers to support employees and guide them towards use of the EAP or else being unaware of the support available to their employees through referral
Utilisation of technology	Lack of utilisation of technology to enable easy access to the services, leading to slow and inefficient manual processes that cannot provide rapid support
Measurement criteria	Traditional measures, such as utilisation of programme, are used as measures of success

*Source*: Adapted from Van Den Bergh 1991; Sonnenstuhl and Trice 2018

Note: Please see the full reference list of the article: Veldsman, D., & Van Aarde, N. (2021). The impact of COVID-19 on an employee assistance programme in a multinational insurance organisation: Considerations for the future. SA Journal of Industrial Psychology/SA Tydskrif vir Bedryfsielkunde, 47(0), a1863. https://doi.org/10.4102/sajip.v47i0.1863, for more information.

Despite all these challenges, EAPs have become a key component of most medium to large employers’ employee value proposition. With the rise of Covid-19, a renewed focus on EAPs have come to the fore as organisations have aimed to provide support to their employees during this period through current EAP solutions. Higher levels of stress, anxiety and fear had a significant impact on the workforce and EAP were utilised to provide a sense of security and support (Couser, Nation, & Hyde, 2020). Because of lockdown restrictions across the world, many employees were faced with the challenge of working remotely, whilst also home-schooling children and having to support sick family members. All these factors have taken their toll, and even though higher levels of employee stress and anxiety have been reported, organisations have not necessarily seen a higher utilisation of current EAPs (Brooks & Ling 2020).

The purpose of this article is to explore the EAP in a South African insurance organisation to better understand the reasons for utilisation before and during COVID-19 and to gain insight into why employees utilise these support services.

### History of the employee assistance programmes under study

The EAP that forms the basis of this study was launched in 2016, in response to the outcomes of an organisational climate survey that identified the need for emotional support for employees. The aim of the EAP included well-being education via monthly newsletters, meal plans, orientation and digital services, as well as a pay-as-you-use offering of counselling and trauma interventions. The employer paid all costs related to the EAP, with employees able to utilise the service free of charge. Employee assistance support was provided through various avenues: telephonic, face-to-face, managerial consultation, trauma debriefing and counselling. The EAP offering allowed self-referral and management referral to services, whilst information and awareness were available without referral, continuously increasing awareness, providing information about the programme and topical issues. To combat the challenges generally associated with EAPs, the organisation and the programme administrators ensured confidentiality and adhered to the relevant professional services’ ethical codes. The organisation drove well-being not only through the EAP but also through its culture, communication, leadership and employee value proposition. The programme has seen steady utilisation since 2016 and frequent awareness campaigns were utilised to help employees understand what services were available and how to utilise these. The organisation enhanced the scope of the EAP in 2018 to include well-being-related interventions through the establishment of an integrated employee value proposition framework as part of the broader human capital strategy. The framework drove compelling workplace experiences through various levers that attract and retain employees and position well-being as critical enablers of employee productivity and experience. The broadening of scope included a shift in the EAP’s focus to more preventative care, whilst also dealing with problems already presenting within the workforce and covered aspects of financial, emotional, spiritual and physical well-being.

At the time of the study, the EAP was well established within the organisation and integrated into a variety of human capital practices.

## Research design

### Research approach and setting

The research explored an in-house EAP utilisation in a South African insurance organisation over 9 months, 01 January 2020–30 September 2020, during the COVID-19 pandemic. The organisation employs *n* = 16 207 employees and has offices in South Africa, Lesotho, Namibia, Botswana, Ghana and the United Kingdom. The focus of the research was on the company’s South African operations (*n* = 10 017 employees), as the EAP was limited to that geographical area. The study evaluated the relevance of the EAP to derive insights pertaining to critical considerations for future practice.

### Research method

**FIGURE 1:**
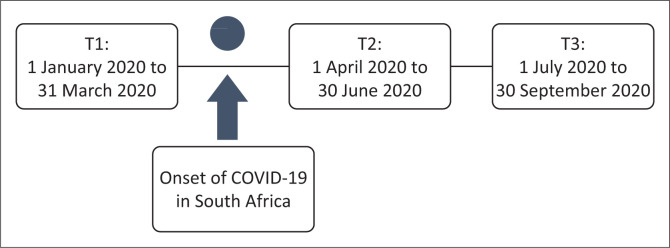
Research time periods.

A qualitative research method was adopted and raw secondary data were obtained from the organisation over the period from 01 January 2020 to 30 September 2020. Data were collected during three designated periods to better understand themes before and after the onset of COVID-19. For this research, the onset of COVID-19 was specified as March 2020, when South Africa went into the first Level 5 lockdown. It was assumed that the impact on EAPs would be most evident once the lockdown period had been announced. Figure 1 indicates the three periods of data gathering according to the pre-and post-lockdown, referred to as T1, T2 and T3.

### Research questions

Within this context, the study aimed to answer the following research questions as it related to the EAP and research setting:

- What are the reasons for employees utilising the EAP?
- Has COVID-19 impacted the reasons why employees utilise the organisational EAP?
- Has COVID-19 resulted in a rise in utilisation of EAP services?

### Description of the sample

The sample consisted of 907 employees who utilised the EAP over a 9-month period, which yielded 1 002 instances where employees utilised the EAP, referred to as cases. The sample’s age distribution was as follows: 196 employees aged 21–30, 376 employees aged 31–40, 226 employees aged 41–50, 82 employees aged 51–60 and 9 employees older than 61; 18 employees did not disclose their age. The gender distribution of the sample was 684 women and 223 men. The marital status of the sample was: 470 single, 323 married, 61 divorced, 7 widowed, 38 identified as ‘Other’, and one separated; 7 chose not to disclose their marital status. To protect the anonymity of the employees who had utilised the programme, no identifying information was recorded.

### Analysis of data

Secondary data, in the form of anonymised case notes, was used for the analysis with the informed consent of participants to utilise the information for research purposes. Ethical standards with regard to the accessing, utilisation and storage of information were followed as outlined by Barnhill and Barnhill (2014). Thematic analysis was utilised to interpret the data aligned with the research question. Thematic analysis refers to the identification, analysis and interpretation of patterns from qualitative data and organising the data into meaningful themes (Terry, Hayfield, Clarke, & Braun, 2017). A five-phase approach was followed as described by Clarke and Braun (2014):

- Phase 1: Familiarisation of the data

This phase entailed the immersive process of working through the detailed case notes and transcripts. For this study, the case notes from the EAP sessions were utilised with permission from participants and the organisation.

- Phase 2: Generation of codes

Coding techniques were applied to identify similarities and patterns within the data across the different cases and points of time. Coding was used to group similarities that serve as input into the identification of themes.

- Phase 3: Searching for themes

Initial themes were developed from the codes identified in phase 2 and further expanded to sub-themes encapsulated in these themes.

- Phase 4: Review

Themes were reviewed in line with the original data set to clarify meaning in relation to the research question.

- Phase 5: Develop naming conventions for the themes

The essence of the themes was described through short statements of meaning.

To control for bias, the researchers also cross-validated identified themes with participants in an attempt to ensure that information was interpreted in a believable manner. As a limitation of qualitative research, generalisability of the information to other areas should be treated with caution.

## Findings

Analysing and comparing the utilisation of the EAP over 9 months, divided into three periods highlighted the key themes. T1, ending 31 March 2020, represents the utilisation of the programme pre-COVID-19, whilst T2 and T3 represent data gathered during the COVID-19 lockdown during the national state of disaster in South Africa. From 01 January 2020 to 30 September 2020, 1002 EAP cases were reported, and 907 employees utilised the EAP, indicating that some employees utilised multiple services or services over multiple reporting periods. Through the coding exercise, the data were grouped into clusters of themes that related to the specific reason why employees sought support through the EAP. Table 3 highlights the different themes extracted from the data:

**TABLE 3:** Overview of themes identified.

Theme	Description
Relationships	This theme described cases related to personal relationship, divorce, marital conflicts and experiences of domestic violence.
Family	The family theme referred to cases that involved family members, child behavioural problems, child educational problems, parental guidance issues, family member illness and extended family issues.
Psychological well-being	The psychological well-being theme referred to instance of depression, panic attacks, life adjustment issues, anger management and psychiatric requirements.
Work related	This theme referred to employee performance issues, adjustments to role or change, managerial challenges, burnout because of work-related pressure, work overload, disciplinary issues and performance challenges.
Trauma	The trauma theme related to incidents such as robbery, hijacking, rape, house breaking and car accidents.
Coronavirus disease 2019 (COVID-19)	This theme referred to instances of fear of self or significant other contracting the disease, dealing with COVID-related anxiety, grief and containment of employees testing positive.
Legal	This theme referred to issues related to legal advice and support.
Health and lifestyle	This theme described instances of health and lifestyle advice, guidance and counselling.
Financial	The financial theme referred to debt counselling, financial advice and basic financial literacy issues.
Addiction	The addiction theme referred to instances of substance abuse and addictive behaviour.
Information	This theme referred to general informational queries where employees utilised the employee assistance programme to gain further knowledge to services that are provided.
Other	This theme was utilised to describe cases that did not fall into the themes above or only occurred once during the period.

Table 4, indicates the utilisation of services per number of cases, using the themes as identified in Table 3. In the context of this study a case refers to an instance where an employee accesses and utilises EAP services.

**TABLE 4:** Frequency of themes per period.

Themes	Cases	%
**T1: January 2020–March 2020**		
Relationships	69	22
Family	58	19
Psychological well-being	55	18
Work-related	42	14
Trauma	33	11
Legal	26	8
Health and lifestyle	12	4
Financial	6	2
Addiction	4	1
Information	2	1
Other	2	1
**Total**	**309**	**100**
**T2: April 2020–June 2020**		
Work-related	57	21
Relationships	34	13
Family	33	12
Psychological well-being	33	12
Coronavirus disease 2019 (COVID-19)	32	12
Health and lifestyle	31	11
Legal	19	7
Trauma	18	7
Information	9	3
Financial	2	1
Other	3	1
**Total**	**271**	**100**
**T3: July 2020–September 2020**		
Family	73	17
Psychological well-being	68	16
Trauma	65	15
COVID-19	63	15
Work-related	52	12
Relationships	45	11
Legal	19	5
Financial	9	2
Information	8	2
Health and lifestyle	8	2
Other	12	3
**Total**	**422**	**100**

The top five themes for T1 were: *Relationships* (22%), *family* (19%), *psychological well-being* (18%), *work-related* (14%) and *trauma* (11%). These themes accounted for 84% of cases for this period, and it was notable that family- and relationship-related matters made up a significant portion of cases in T1. *Work-related* (21%), *relationships* (13%), *family* (12%), *psychological well-being* (12%) and *COVID-19* (12%) were the top five themes for T2, indicating the emergence of COVID-19-related cases. For the purposes of the study, COVID-19-related cases were instances where support was sought as a direct result of the lockdown during COVID-19. T3 showed a significant increase in the number of employees utilising the EAP compared with T1 and T2. The leading themes in T3 were *family* (17%), *psychological well-being* (16%), *trauma* (15%), *COVID-19* (15%), and *work-related* (12%).

Throughout the three periods, *family, psychological well-being*, and *work-related* were the most prevalent themes, indicating that psychological and mental well-being remained critical to employee well-being in managing work demands. COVID-19 emerged as a theme in T2 (12% of cases), and, in T3, 15% of cases indicated a need for the EAP to provide COVID-19-related support.

The themes depicted in Table 5 indicate a shift in the type of cases related to the themes at a sub-theme level. *Family*, although relatively consistent, showed that, post-lockdown, more cases related to parental guidance and family member illness occurred. This may be as a result of parents being confronted with home-schooling children (who were unable to attend school) whilst working from home, as well as the effects of COVID-19 on families, with employees battling to cope with the uncertainty of supporting family members through the disease. The sub-themes of COVID-19 shifted from *fear of testing positive* and *testing positive* as the most frequent cases in T2 to *testing positive* and *anxiety* in T3, indicating changes in the support required.

**TABLE 5:** Top five themes and sub-themes per period.

Theme	T1			T2			T3		
	Rank	Sub-theme	Cases	Rank	Sub-Theme	Cases	Rank	Sub-theme	Cases
Relationships	1	Personal relationship	30	2	Personal relationship	17	-	-	-
		Divorces	15		Marital conflicts	6		-	-
		Marital conflicts	11		Divorces	5		-	-
		Domestic violence	5		Domestic violence	3		-	-
Family	2	Family member-related	23	3	Family member related	16	1	Family member-related	36
		Child behavioural problems	8		Parental guidance	6		Child behavioural problems	14
		Child behavioural/ educational	7		Child behavioural problems	3		Family member illness	10
		Extended family issues	7		Extended family issues	3		Child behaviour/education	4
Psychological well-being	3	Depression	40	4	Depression	23	2	Depression	37
		Panic attacks	6		Phase of life/adjustment issues	5		Phase of life/adjustment issues	15
		Phase of life/adjustment issues	5		Panic attacks	3		Panic attacks	7
		Anger management	2		Anger management	1		Psychiatric assessment required	3
Work related	4	Performance issues	10	1	Managerial consultancy	8	5	Burn out	7
		Adjustment to change in personal work role	4		Performance issues	8		Work overload	7
		Performance management	4		Lack of focus/concentration	6		Conflict with manager	5
		Work performance	3		Disciplinary issues	5		Lack of focus/concentration	5
Trauma	5	Personal	11	-	-	-	3	Personal	40
		Robbery	7		-	-		Robbery	5
		Hijacking	4		-	-		House breaking	4
		Rape	4		-	-		Car accident	3
Coronavirus disease 2019 (COVID-19)	-	-	-	5	Fear of self or significant other contracting	11	4	Tested Positive	28
		-	-		Tested positive	11		Anxiety	11
		-	-		Poor coping skills	3		Fear of self or significant other contracting	6
		-	-		COVID-19-related grief	2		Poor coping skills	5

The theme *psychological well-being* remained relatively similar across periods; however, it is notable that *depression* consistently represented most cases within this theme, evidencing the need for psychological services as part of the EAP. Within the context of this theme, however, it should be observed that the perception that the EAP focuses only on psychological or counselling-related services could have led to the high prevalence of these type of cases, as employees were not always aware of the ‘non-traditional’ services offered by the programme. The *work-related* theme showed the most movement in terms of the sub-themes representing the type of support required by employees. *Work-related* cases predominantly centred on *performance* in T1, whilst the cases in T2 reflected *lack of focus/concentration* and *disciplinary issues* as new themes. The change in cases might be attributable to the change associated with the lockdown and the reality of COVID-19 as managers adapted to managing remotely and employees adapted to working remotely. T3 showed *burnout* and *workload* as the top two sub-themes, illustrating possible work strain and the effects of working and the disintegration of traditional work/home boundaries. *Trauma* was in the top five themes for T1 and T3, with similar sub-themes as *Personal*. For T1 to T2, there seemed to be a strong initial rise in *health and lifestyle*-related issues; however, this trend seemed to return to similar levels than experienced previously in T3. This could indicate some initial struggles with employees adapting to work boundaries whilst working remotely and not balancing work and family life. With regard to *trauma*, it is evident that, whilst there was an initial decrease in trauma-related issues between T1 and T2, a significant rise in cases was evident in T3. This could have been because of the initial lockdown restricting movement in T2 and a decrease in trauma-related incidents such as robbery, hijacking and car accidents. In T3, however, this situation seemed to change, with a significant increase in personal trauma-related incidents, which could be because of employees returning to the workplace whilst also starting to commute and move around more freely under the relaxed lockdown conditions. As expected, the incidence of financial matters remained consistent, given the fact that the organisation continued to pay full salaries to all employees during lockdown.

Table 5 provides a summary of the top five sub-themes related to the primary themes described here. Even though some consistency exists in terms of the top five primary themes that manifested, there seem to be differences in priority and number of cases identified across the three time periods. As expected, COVID-19-related matters were more prevalent in T2 and T3, because of the rise in uncertainty, the increased number of positive cases and the impact of lockdown on the workforce.

## Discussion

A significant change in the number of cases was observed across the three time periods. In T2, which was immediately after the lockdown was announced, the number of cases declined, yet a significant increase in T3 led to the conclusion that the impact of COVID-19 on the holistic well-being of employees was still in its infancy, and it is expected that more cases directly related to or associated with COVID-19 will arise in future.

It seems that the themes of *psychological well-being, family, relationships* and *work-related* matters remained prevalent throughout the three-time periods. The decline of trauma-related matters in T2 was ascribed to employees’ reduced physical movements under lockdown Level 5’s restrictions. The impact of COVID-19 is evident, not only in COVID-19-related cases but also in the rise in *family, work-related* and *psychological well-being* cases in T3. This trend indicates that the impact of COVID-19 will be far-reaching, beyond just the immediate impact of loss of life and inability of employees to be productive whilst ill. Surprisingly, no increase in domestic violence cases was identified from T1 to T3, despite the national increase in gender-based violence cases (Shoba, 2020). However, it is known that these cases often remain unreported, and the lack of visible cases does not imply that this issue is not prevalent in the organisation and the situation should be monitored closely going forward. The data for T3 also indicate that burnout and managerial conflict are becoming prevalent from a work-related perspective. This could indicate that the impact of remote work will significantly impact the workforce in the future and that workload needs to be monitored and planned differently. Managerial skills and styles will also come under scrutiny with the changing requirements of managing a hybrid or even fully remote workforce. Mental well-being, specifically depression and anxiety, occurred consistently across all three time periods, which will remain an important consideration for employers.

The study set out to answer the following research questions:

### Research Question 1: What are the reasons for employees utilising the employee assistance programme?

The study found that employees utilised the EAP for a variety of reasons. The study identified 12 themes that describe the reasons why employees utilised the programme. The themes of *relationships, family, psychological well-being, work-related, trauma, legal, health and lifestyle, financial, addiction, information* and *other* were found consistently across all three time periods. For T2 and T3, an additional theme specifically related to COVID-19 reasons was also identified.

### Research Question 2: Has COVID-19 impacted the reasons why employees utilise the organisational employee assistance programme?

The study showed some consistency with regard to the reasons why employees utilised the EAP, even after the onset of the pandemic. As a result of COVID-19, additional reasons emerged with regard to why employees utilised the EAP. The study also showcased that at a sub-theme level, there was some consistency of the types of reasons why employees utilised the EAP across all three time periods. The prevalence of cases however differed, and this could be because of external circumstance because of the pandemic, for example, less cases related to hijackings in T2 and T3 as a result of limited commuting because of lockdowns.

### Research Question 3: Has COVID-19 resulted in a rise in utilisation of support services?

The data showed a decline in utilisation in T2, followed by an increase in T3. This may be because of employees focussing inward, adjusting and coping with the immediate change associated with the Level 5 lockdown. This could also imply that employees believed that the impact of COVID-19 would be temporary, and, within the first 3 months, still tried to cope with the immediate changes that working from home entailed. There was a consistent increase in the utilisation of support services related to COVID-19 in T3, with cases increasing by almost 98%. Therefore, it can be concluded that COVID-19 resulted in an increased need and utilisation of support services in both COVID-19-specific and other related cases. It can also be concluded that COVID-19 could potentially give rise to more cases, given the impact that COVID-19 will have on the South African economy and the additional stress and anxiety that employees need to deal with as a result.

## Recommendations for future research

The study confirmed that the need for services is increasing steadily as employees adjust to life and work in a COVID-19 world. EAPs need to be aligned to employees’ changing requirements dealing with the demands and additional strain brought forth by the pandemic. Services related to psychological and mental well-being remain critical, yet an increase in services related to trauma and dealing with family issues and associated responsibilities need to be enhanced. The ability of the EAP to address potential cases of burnout also requires additional focus, through preventative education and equipping leaders and managers to identify cases of burn-out.

From a practical perspective, the study highlights the need to relook the EAP scope whilst also building on the current services offered to employees. Administrators of the EAP should partner with other service providers to ensure that a broader scope of services is made available to employees. The utilisation of the EAPs remains low, which could indicate that employee education and manager training are not effective in helping employees to understand the role of the EAP in their well-being. As highlighted by the literature, this could indicate that the stigma associated with EAPs is still evident and that organisations will have to focus on promoting use of the EAP and educate employees regarding its value in the workplace.

## Limitations

The study focused on only one organisation in the South African context; as such, the generalisability of the findings is limited. The study also only focused on three points in time over a 9-month period. Future studies should be extended to include more data over time, especially as South Africa enters additional waves of COVID-19. The study should also be repeated in other organisations, to determine if the findings are similar for organisations of different sizes and in other industries. As the sample of this study consisted of a predominantly professional services workforce, it is recommended that the study be replicated in organisations with more blue-collar employees, to evaluate how the utilisation of an EAP changes over time in such a context.

## Conclusion

The purpose of this study was to explore the utilisation and relevance of an EAP at three designated points in time before and after COVID-19 lockdown in a multinational insurance organisation. The study found significant changes in how the EAP was utilised in these periods, as well as significant variations in the volume and types of cases. The study also reflected on the literature related to EAPs and the limitations currently inhibiting the effectiveness of EAPs. In conclusion, it is recommended that going forward organisations should increase the scope of their EAP and adopt a holistic approach to well-being, one that goes beyond the parameters of traditional wellness programmes and incorporates services specific to dealing with the difficulties arising from the pandemic.
